# Cost-effectiveness of selective laser trabeculoplasty as a replacement for hypotensive eye drops in the Brazilian public health system

**DOI:** 10.1016/j.clinsp.2025.100650

**Published:** 2025-04-23

**Authors:** Leopoldo Ernesto Oiticica Barbosa, Wilma Lelis Barboza, Ricardo Agusto Paletta Guedes, Alfredo Chaoubah, Marcelo Hatanaka

**Affiliations:** a Universidade de São Paulo, São Paulo, SP, Brazil; b Instituto de Olhos de Maceió, Farol, Maceió, AL, Brazil; c Universidade Federal de Juiz de Fora, Juiz de Fora, MG, Brazil

**Keywords:** Glaucoma, Eye drops, Intraocular pressure, Cost-effectiveness

## Abstract

**Objective:**

This study aimed to determine the cost-effectiveness of selective laser trabeculoplasty (SLT) as a replacement for hypotensive eye drops in the Brazilian Unified Health System (SUS).

**Methods:**

The authors conducted a cost-effectiveness economic assessment using data from a previously published prospective, non-randomized, single-arm interventional study. The costs were estimated from the perspective of the SUS, using data from the SIGTAP. The authors proposed a value of R$ 588.00 for bilateral SLT, based on the observed cost reduction with medications in the SLT group. Effectiveness was assessed as a reduction in intraocular pressure (IOP).

**Results:**

The average annual cost of medications per patient was R$ 669.53 in the reference group (continuous use of eye drops) and R$ 80.62 in the intervention group (SLT). The average IOP reduction was 9.3 mmHg in the reference group and 9.8 mmHg in the intervention group. SLT proved to be a dominant strategy compared to the continuous use of eye drops, presenting a greater reduction in IOP and a lower total annual cost. The incremental cost-effectiveness ratio (ICER) was negative (-R$ 1.82), indicating that SLT is more effective and less costly.

**Conclusions:**

SLT is a cost-effective alternative to hypotensive eye drops in the treatment of glaucoma in the SUS. The adoption of SLT can optimize resource allocation and improve clinical outcomes for patients with glaucoma. Implementing SLT at the proposed procedural cost optimizes spending by substantially reducing long-term medication costs and offering a sustainable alternative within the healthcare system.

## Highlights

• SLT cost-effective for glaucoma in SUS.
• SLT reduces costs & improves outcomes.
• Economic analysis of SLT in Brazil.
• SLT as alternative to eye drops.

## Introduction

Glaucoma is a chronic condition and the major cause of irreversible blindness globally, placing a growing strain on healthcare systems, particularly for patients affected by this disease or those at risk requiring long-term treatment. With an increase in the aging population, approximately 111.8 million individuals are expected to develop glaucoma by 2040.^1^

Randomized clinical trials reveal that lowering Intraocular Pressure (IOP) is key to slowing the progression of visual field loss linked to Open-Angle Glaucoma (OAG).^2^, ^3^, ^4^, ^5^ The main IOP control options are topical medications, laser trabeculoplasty, and glaucoma surgeries like trabeculectomy.

Topical therapy has long been the mainstay of glaucoma treatment, with various classes of medications developed to lower IOP through different mechanisms of action. However, recent evidence supports a paradigm shift toward Selective Laser Trabeculoplasty (SLT) as an initial or adjunctive therapy for primary OAG. ^6^ The shift is driven by issues like high rates of nonadherence to medication, potential side effects linked to drugs, their impact on patients’ quality of life, and cost.^7^

In Brazil, glaucoma treatment in the public health system follows the Clinical Protocol and Therapeutic Guidelines, recommending antiglaucomatous eye drops as the first therapeutic option. However, drug treatment has side effects and imposes substantial costs on patients and the healthcare system. Since OAG is a chronic condition, long-term medication use can be financially burdensome. Thus, cost-effective non-pharmacological approaches like SLT have gained recognition as viable alternatives.^8^

A recent clinical study conducted in the Brazilian population evaluated SLT's effectiveness as an alternative to eye drops for glaucoma treatment. Eye drops were initially discontinued, and SLT was administered as the initial treatment. Subsequently, eye drops were reintroduced, if necessary, according to the National Commission for the Incorporation of Technologies (CONITEC) guidelines. While the study did not focus on the economic analysis, it showed stable IOP after one year of follow-up and reduced reliance on eye drops for disease control. These results underscore SLT's potential as an effective long-term glaucoma treatment.^9^

Economic analysis is vital for guiding healthcare choices, from resource allocation by communities and healthcare systems to choices made by patients and their physicians. Thus, understanding whether a treatment option is cost-effective is crucial for various stakeholders, including health policy decision-makers, third-party payers, ophthalmological professionals, and patients. As healthcare costs rise, identifying ways to reduce expenses while maintaining treatment quality is essential.

This study aimed to determine whether SLT is a cost-effective alternative to eye drops in the Brazilian public health system using real clinical data from the Brazilian population.

## Methods

A cost-effectiveness economic assessment was conducted using data from a previously published prospective, non-randomized, single-arm interventional study by the same authors.^9^ The clinical outcomes from this study formed the basis for comparing eye drop usage, proposed intervention cost, and the SLT application. The study protocol was reviewed and approved by The Ethics Committee of Hospital das Clínicas of the University of São Paulo Medical School. The study protocol number is 4.144.552. All study participants provided written informed consent before participation. All study participants provided written informed consent before participation. The study was registered on ensaiosclinicos.org.br with the Universal Trial Number U1111–1255–1601. The authors followed the STROBE (Strengthening the Reporting of Observational Studies in Epidemiology) guidelines to ensure transparent and comprehensive reporting of this observational study. Below is a summary of the clinical study methods used for cost-effectiveness analysis.

Inclusion criteria for the study were patients > 40 years of age with mild to moderate OAG (defined perimetrically as an MD better than −12 dB, with no threat to fixation, and no points with 0 dB sensitivity in the central 5°) who were already using hypotensive eye drops. Exclusion criteria included patients with advanced glaucoma (defined as MD worse than −12 dB or with a threat to fixation)^10^ patients previously submitted to incisional or laser glaucoma surgery, intraocular inflammation within the last 3 months, ocular trauma or surgery in the past 6 months, or corneal alterations affecting IOP measurement.

Following selection, the participants underwent a washout period for their glaucoma medications, which differed according to the class of hypotensive drugs used. This included four weeks for beta-blockers and prostaglandin analogs, two weeks for adrenergic agonists, and five days for cholinergic agonists and carbonic anhydrase inhibitors.

Following the medication washout period, the IOP of the participants was measured, referred to as the baseline IOP. The patients were then followed up for one year, and a personalized target IOP was calculated as a 25 % reduction from the baseline IOP, rounded to the nearest mmHg or 21 mmHg, whichever was lower. Hypotensive medication was added if the mean IOP exceeded the target IOP at any visit starting from month 3, following the Clinical Protocol and Therapeutic Guidelines for glaucoma of the National Commission for the Incorporation of Technologies in the SUS (CONITEC).^11^ Medications were introduced in the following sequence (observing contraindications): 1st line (Timolol maleate), 2nd line (Dorzolamide, Brimonidine, or Brinzolamide), and 3rd line (prostaglandin analogs).

The authors conducted a cost analysis from the perspective of the Brazilian Unified Health System (SUS) based on the proposed treatment changes, considering the guidelines established by the Ministry of Health. The authors used the Procedure Table Management System (SIGTAP)^12^ as a reference to estimate the direct medical costs, which is widely adopted by the SUS as a funding agent for all reimbursement values. However, notably, a specific value for Selective Laser Trabeculoplasty (SLT) is unavailable in SIGTAP. Thus, establishing a viable value that allows for resource reallocation without increasing costs for the payer is necessary.

To address this gap, the observed reduction in medication costs was utilized to propose a feasible value for the SLT procedure, ensuring that its implementation would not impose additional financial burdens on the public health system. This was particularly important as no specific reimbursement value for SLT currently exists within the SUS framework. The estimation of the SLT cost in this study followed an adapted micro-costing approach, grounded in observational data and economic reasoning. This methodology, commonly employed in economic analyses, is particularly effective for allocating costs in contexts where comprehensive data on individual cost components ‒ such as infrastructure, consumables, and labor ‒ are unavailable.^13^

## Data source

The calculation was based on the observed reduction in costs associated with hypotensive eye medications, which were replaced by SLT in the intervention group. These costs were derived from reimbursement tables widely used by the SUS, including SIGTAP. This provided a robust and realistic foundation for the estimation process, ensuring alignment with real-world data.

## Calculation methodology

- Initially, the annual per-patient medication costs were calculated for the reference group.

- Subsequently, the costs of bilateral SLT procedures were projected based on the savings generated from reduced medication use after the intervention.

- The value of R$ 588.00 for bilateral SLT was designed to maintain parity between the annual costs of the intervention and reference groups, ensuring that SLT could be implemented without increasing the overall financial burden on SUS.

## Theoretical support

This approach aligns with established guidelines for economic analyses in resource-constrained settings, as outlined by the Brazilian Ministry of Health and the CONITEC. By reallocating the savings from reduced medication usage to fund SLT, this method exemplifies an efficient use of scarce resources, a widely accepted principle in public health systems worldwide.^14^

### Recognized limitations

While this approach provides a practical and realistic cost estimate, it is important to acknowledge its limitations. A full micro-costing study could offer more precise insights by accounting for factors such as infrastructure, equipment maintenance, professional training, and other indirect costs. Future analyses incorporating these elements would refine the proposed values and offer a more comprehensive understanding of the true cost of SLT.

The costs considered in this study include direct medical expenses associated with the treatment provided by Reference Centers for Glaucoma Treatment within the SUS, encompassing both medication and SLT application. However, direct and indirect non-medical costs were not included in this analysis.

The research horizon was for 1-year.

Furthermore, the economic analysis compared two groups:

1. Reference Group (Baseline Case): A cohort of patients using anti-glaucoma eye drops with projected effectiveness and costs found before the intervention (SLT application) over one year of follow-up, assuming they continued with the same baseline treatment.

2. Intervention Group: A cohort of patients after SLT application as a substitute therapy for eye drops, examining the effectiveness and real costs of SLT application and the requirement to reintroduce anti-glaucoma eye drops over one year of follow-up.

The decision to use a one-year time horizon for the economic analysis was guided by the availability of clinical data from the prospective study that formed the foundation of this work, which included a 12-month follow-up. This timeframe was chosen to allow for a thorough evaluation of SLT's impact on costs and its effectiveness in reducing IOP, while staying within the practical boundaries of the study's design.

Effectiveness was assessed as a reduction in IOP (mmHg). This measurement was obtained for the reference group by subtracting the medicated baseline IOP (before washout) from the peak IOP at baseline after medication washout. IOP was calculated in the intervention group by subtracting the medicated or non-medicated IOP at 12 months after the peak IOP after medication washout at baseline. Thus, pressure-lowering capacity was obtained from the same baseline value in both groups.

The Incremental Cost-Effectiveness Ratio (ICER) was obtained by calculating the average cost required to achieve a 1 mmHg reduction in IOP with each therapeutic alternative (ICER = R$ / mmHg).

Additionally, the authors analyzed the cost impact and potential savings generated by adopting SLT as a substitute therapy for medication in glaucoma treatment centers within the SUS.

A univariate sensitivity analysis was performed by varying the average cost of each alternative using the lower and upper limits of the 95 % Confidence Interval to test the robustness of the economic analysis.

## Results

Table 1 lists the unit costs for each healthcare resource considered in the cost analysis.

**Table 1:** Health resources and their respective unit costs.

Resources	Unit Cost (R$)
First-line hypotensive eye drops (Timolol Maleate) ‒ Binocular	18,66
2nd line hypotensive eye drops (Dorzolamide or Brimonidine or Brinzolamide) ‒ Binocular	79,38
3rd line hypotensive eye drops (Prostaglandin Analogs) ‒ Binocular	127,98

Source: SIGTAP Table.

**Fig. 1:**
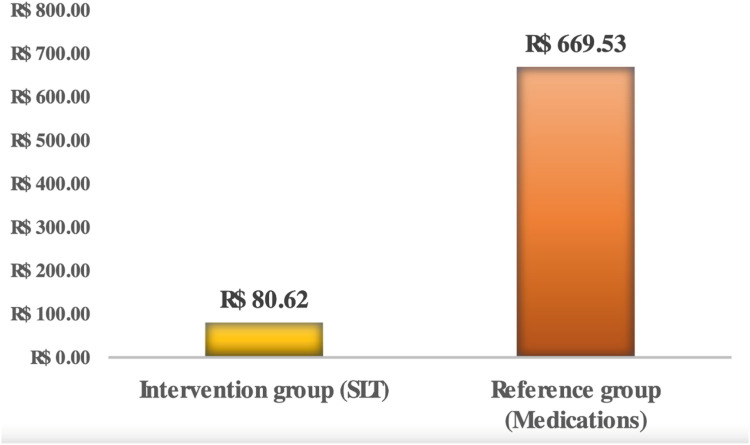
Average annual cost of medications per patient in each group (R$).

The mean ± standard deviation number of medications per patient at the end of one year was 2.26 ± 1.06 and 1.02 ± 0.88 in the reference and intervention groups, respectively, generating an average cost (95 % CI) of R$ 669.53 (R$ 586.39 – R$ 752.65) and R$ 80.62 (R$ 48.31 – R$ 112.91), respectively, for each group (Fig. 1). The authors propose a value of R$ 588.00 for the bilateral SLT procedure based on the observed cost reduction with medications in the SLT group (Fig. 1). This value was proposed to maintain the total treatment cost similar to that of the reference group (continuous use of eye drops), ensuring efficient reallocation of available resources and economic viability of the healthcare system.

**Fig. 2:**
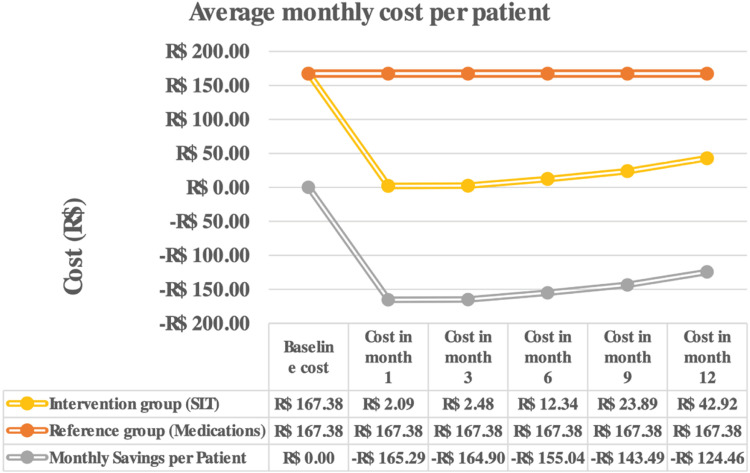
Evolution of the average cost and cost savings generated per patient with each monthly therapeutic strategy.

Fig. 2 illustrates the evolution of the average cost per patient over the follow-up period and the savings generated by applying SLT. The average effectiveness was 9.3 mmHg and 9.8 mmHg, respectively, in the Reference (medications) and Intervention Groups (SLT).

Table 2 presents the cost-effectiveness analysis, including the suggested value of the SLT for implementation in the SUS. The sensitivity analysis, which adjusted the average treatment costs, did not change the results, as the average annual cost for the reference group remained higher, and the effectiveness remained lower than that of the intervention group. The negative ICER value (-R$ 1.82) indicates that SLT is a dominant strategy compared to continuous medication use. Dominance in economic analysis occurs when an intervention is both more effective (greater IOP reduction) and less costly (lower total annual cost). This reinforces the conclusion that the SLT intervention is dominant (strong dominance) over continuous medication use in SUS glaucoma treatment centers. This result underscores the dual advantage of SLT in achieving better clinical outcomes while reducing overall healthcare expenditures within the SUS framework.

**Table 2:** Cost-effectiveness analysis.

Cost-effectiveness analysis				
	Cost (R$)^a^	Effectiveness (mmHg)^b^	Cost / Effectiveness (R$ / mmHg)^c^	ICER^d^
Intervention group (SLT)	668,62^e^	9.8	68,22	-R$ 1,82
Reference group (medication)	669,53	9.3	71,99	

SLT, Selective Laser Trabeculoplasty.

a Average annual cost per patient.

b Average pressure reduction capacity.

c Cost for each 1 mmHg of IOP reduction.

d Incremental Cost-Effectiveness Ratio, obtained by dividing the difference in costs by the difference in effectiveness: ICER = (668,62 – 669,53) / (9,8 – 9,3).

e Average annual cost of reintroducing ocular hypotensive medications added to the suggested value of SLT application (bilateral).

## Discussion

Herein, SLT was found to be dominant over the usual medication therapy in patients with mild-to-moderate OAG within the context of the glaucoma reference centers of the SUS over one year. According to Ministry of Health guidelines^15^ cost-effectiveness studies are crucial for guiding decisions by managers, physicians, and patients. These studies compare the costs and effects of different therapeutic alternatives, achieving the same clinical outcomes but with various magnitudes. In this analysis, IOP reduction was used as the effectiveness criterion. The results are expressed as the ICER, which measures the cost per unit of additional benefit. Cost-effectiveness evaluations can categorize interventions as more expensive and effective, more expensive and less effective, cheaper and more effective, or cheaper and less effective.^16^ An alternative is deemed “dominated” and typically rejected if it is more expensive and less effective, whereas it is deemed “dominant” and preferred if it is cheaper and more effective, as SLT was in the present findings. Moreover, these findings indicate that with improved resource reallocation, SLT is cost-effective and economically viable. Implementing SLT at the proposed procedural cost optimizes spending by substantially reducing long-term medication costs and offering a sustainable alternative within the healthcare system.

The LiGHT study^6^ demonstrated that using SLT in newly diagnosed patients is more economically advantageous, leading to lower disease progression rates and a reduced requirement for surgical interventions over six years. Introducing SLT only for new cases within the SUS could gradually reduce costs in the long term as more patients are diagnosed. However, this study suggests that even patients already in the eye drop distribution program could benefit from substituting these medications with SLT. This substitution was followed by the gradual reintroduction of eye drops as required according to the Clinical Protocol and Therapeutic Guidelines (PCDT).^17^

Herein, a main aspect of the cost-effectiveness analysis was using medications based on the cost criteria, as recommended by CONITEC. This resulted in the substitution of the most expensive third-line medications with equally effective technologies for lowering IOP.^18^, ^19^, ^20^, ^21^, ^22^, ^23^ Data from DATASUS in 2023[25] show that 71.5 % of patients registered in the government's eye drop distribution program use third-line medications. Given these findings, incorporating SLT into PCDT could considerably reduce costs and improve the allocation of already limited resources.

A limitation of this study is the lack of a detailed micro-costing analysis to accurately determine the true value of implementing SLT. The most precise method would account for factors like physical infrastructure, staff, materials, equipment depreciation, and operational costs, such as energy and maintenance. However, the authors chose a simpler approach and compared it to the resources already used in the treatment with eye drops. Though this comparison offers a practical estimate, a micro-costing study might reveal a higher value for SLT, potentially increasing the financial burden on the payer and making technology adoption more expensive. Additionally, the lack of a specific payment code for SLT in the SUS hinders an accurate assessment of its cost-effectiveness.

Another considerable limitation of the economic analysis is the exclusion of adherence to clinical treatment. Studies have shown that the self-reported adherence rate to glaucoma treatment is low, ranging from 30 % to 80 %.^24^^,^^25^ This can substantially increase the risk of disease progression and associated treatment costs due to the requirement for more medications and surgeries^26^^,^^27^ Including this factor could substantially change the results of cost-effectiveness, as a treatment independent of patient adherence, such as SLT, could prove to be more economically advantageous by reducing the frequency of more invasive and costly long-term medical interventions.

The choice of a one-year time horizon for the economic analysis was based on the availability of clinical data from the prospective study underpinning this work, which included a 12-month follow-up. This timeframe allowed for a focused evaluation of SLT's impact on costs and effectiveness in reducing IOP, considering the expenditures associated with eye drop use in the reference group.

However, the authors acknowledge that glaucoma is a chronic condition requiring lifelong management. Previous studies, such as the LiGHT Trial^6^, ^8^ suggest that the clinical and economic benefits of SLT could be further amplified over longer time horizons due to cumulative reductions in medication use and slower disease progression. Modeling studies projecting outcomes over 5–10 years could provide a more comprehensive understanding of the long-term sustainability and cost-effectiveness of SLT. Such analyses would be particularly relevant in assessing the financial and clinical implications of incorporating SLT into chronic disease management strategies in public health systems.

“While SLT offers clear economic and clinical benefits, its practical implementation within the SUS comes with several challenges. These include ensuring that ophthalmologists receive specialized training, acquiring and maintaining laser equipment, and establishing the necessary infrastructure in public health centers. Additionally, significant regional disparities in healthcare access and cost variations across different parts of Brazil could create barriers to uniform adoption. Addressing these obstacles will require coordinated efforts, including investments in training programs, infrastructure development, and strategies to ensure equitable access to SLT across all regions. Such measures are essential to fully realize the clinical and economic potential of SLT as a sustainable treatment option in the SUS and to assist in determining the ideal cost of SLT within the SUS, given the absence of this value in the current pricing table”.

In conclusion, implementing SLT in the Brazilian public health system could bring substantial benefits to patients by reducing medication use, minimizing side effects, and improving treatment adherence. For payers, it would lead to more efficient resource allocation, as the total treatment cost would be reduced compared with the continuous eye drop use. Adopting the suggested minimum value for SLT would ensure its economic viability within SUS while maintaining clinical effectiveness and optimizing the resources available for glaucoma treatment.

## Declaration of competing interest

The authors declare no conflicts of interest.
